# Circadian Kinetics of Cell Cycle Progression in Adult Neurogenic Niches of a Diurnal Vertebrate

**DOI:** 10.1523/JNEUROSCI.3222-16.2017

**Published:** 2017-02-15

**Authors:** Veronica Akle, Alexander J. Stankiewicz, Vasili Kharchenko, Lili Yu, Peter V. Kharchenko, Irina V. Zhdanova

**Affiliations:** ^1^Department of Anatomy and Neurobiology, Boston University School of Medicine, Boston, Massachusetts 02118,; ^2^School of Medicine, Universidad Los Andes, Bogota, 111711, Colombia,; ^3^Harvard-Smithsonian Center for Astrophysics, Harvard University, Cambridge, Massachusetts 02138,; ^4^Department of Biomedical Informatics, Harvard Medical School, Boston, Massachusetts 02115, and; ^5^Harvard Stem Cell Institute, Cambridge, Massachusetts 02138

**Keywords:** adult neurogenesis, BrdU, cell cycle, circadian, S phase, zebrafish

## Abstract

The circadian system may regulate adult neurogenesis via intracellular molecular clock mechanisms or by modifying the environment of neurogenic niches, with daily variation in growth factors or nutrients depending on the animal's diurnal or nocturnal lifestyle. In a diurnal vertebrate, zebrafish, we studied circadian distribution of immunohistochemical markers of the cell division cycle (CDC) in 5 of the 16 neurogenic niches of adult brain, the dorsal telencephalon, habenula, preoptic area, hypothalamus, and cerebellum. We find that common to all niches is the morning initiation of G_1_/S transition and daytime S-phase progression, overnight increase in G_2_/M, and cycle completion by late night. This is supported by the timing of gene expression for critical cell cycle regulators cyclins D, A2, and B2 and cyclin-dependent kinase inhibitor p20 in brain tissue. The early-night peak in *p20*, limiting G_1_/S transition, and its phase angle with the expression of core clock genes, *Clock1* and *Per1*, are preserved in constant darkness, suggesting intrinsic circadian patterns of cell cycle progression. The statistical modeling of CDC kinetics reveals the significant circadian variation in cell proliferation rates across all of the examined niches, but interniche differences in the magnitude of circadian variation in CDC, S-phase length, phase angle of entrainment to light or clock, and its dispersion. We conclude that, in neurogenic niches of an adult diurnal vertebrate, the circadian modulation of cell cycle progression involves both systemic and niche-specific factors.

**SIGNIFICANCE STATEMENT** This study establishes that in neurogenic niches of an adult diurnal vertebrate, the cell cycle progression displays a robust circadian pattern. Common to neurogenic niches located in diverse brain regions is daytime progression of DNA replication and nighttime mitosis, suggesting systemic regulation. Differences between neurogenic niches in the phase and degree of S-phase entrainment to the clock suggest additional roles for niche-specific regulatory mechanisms. Understanding the circadian regulation of adult neurogenesis can help optimize the timing of therapeutic approaches in patients with brain traumas or neurodegenerative disorders and preserve neural stem cells during cytostatic cancer therapies.

## Introduction

The seminal discovery that the adult brain produces new neurons capable of integrating into existing neuronal circuitry (Altman and Das, 1965; Kaplan and Hinds, 1977; Paton and Nottebohm, 1984) raised hopes that adult neurogenesis could help prevent and treat neurodegenerative and age-dependent brain conditions. However, efficacy of prospective treatments might significantly depend on their timing, if the integrating system of the endogenous circadian clock regulates the cell division cycle (CDC) in neuronal stem and progenitor cells. This is likely since tissue-specific clock-controlled genes are serving as key regulators of cell proliferation in diverse tissues (Fu and Kettner, 2013), and results of *in vitro* studies revealed clock coupling and modulation of cell proliferation in mammalian cells (Beiler et al., 2014; Janich et al., 2014; Feillet et al., 2015).

Whole animal models can further enhance understanding of the role of clock-controlled intrinsic endocrine and physiological factors that define daily changes in the immediate cell environment. The translational value of such models would primarily depend on their temporal adaptation being similar to humans. This is because the core clock molecular mechanisms, including gene expression patterns, daytime suprachiasmatic nuclei neuronal activation, or nighttime surge in circulating melatonin, are highly conserved. In contrast, the downstream clock-controlled functions in diurnal and nocturnal species have a 12 h phase difference. This includes numerous behavioral, cognitive, genomic, enzymatic, metabolic, and neuronal processes that occur in antiphase in, for example, nocturnal mice and diurnal humans. Moreover, the nature of CDC and its high-energy demands suggests that prominent circadian organization of the sleep–wake and feeding cycles can be critical for the overall success of adult neurogenesis (Yamaguchi et al., 2013; Lee et al., 2014; Mueller et al., 2015). Thus, while considering a role for such periodic physiological functions in the production, survival, and incorporation of new neurons into existing networks, translational goals favor diurnal species.

This report, to our knowledge, is the first one to address circadian control of CDC progression in neurogenic niches of a diurnal vertebrate. Previously, the circadian patterns of adult neurogenesis were explored in nocturnal species. Some indicated an increased number of S-phase cells at day–night transition in the brains of lobsters, mice, and rats (Goergen et al., 2002; Guzman-Marin et al., 2007; Bouchard-Cannon et al., 2013). Genetic manipulations of the molecular circadian clock were also found to disrupt cell proliferation in mice (Bouchard-Cannon et al., 2013; Rakai et al., 2014). Other studies, however, did not document daily variation in S phase in nocturnal rodents (Ambrogini et al., 2002; Holmes et al., 2004; Kochman et al., 2006; van der Borght et al., 2006), even when mitosis peaked at night (Tamai et al., 2008).

Our choice of a diurnal vertebrate, the zebrafish, to study circadian control of adult neurogenesis in a whole animal is based on its robust circadian clock (Cahill, 1996; Whitmore et al., 1998), daytime feeding (Peyric et al., 2013), and prominent sleep–wake cycle (Zhdanova et al., 2001). Importantly, this species has remarkably active adult neurogenesis (Zupanc et al., 2005). Each day, thousands of cells in 16 neurogenic niches of the adult zebrafish brain are undergoing division, with the majority of newborn cells eventually differentiating into specialized neurons (Zupanc et al., 2005; Adolf et al., 2006; Grandel et al., 2006; Kaslin et al., 2009, 2013).

Here we demonstrate circadian kinetics of CDC in neurogenic niches of an adult diurnal vertebrate and its enhancement by the entrainment to the environmental light–dark cycle. The pattern common to different neurogenic niches includes transition from G_1_ to S phase of CDC early in the day, with evening peak in the number of cells undergoing DNA replication, and nighttime transition through G_2_/M phases completed by early-morning hours. The magnitude of circadian variation, phase angle of entry into S phase, and the mean S-phase length differ between the five neurogenic niches studied. Together, this suggests the role for both systemic and niche-specific factors in the temporal pattern of adult neurogenesis in a diurnal vertebrate.

## Materials and Methods

### 

#### 

##### Animals.

Adult male zebrafish (*Danio rerio*, wild-type AB strain), 12 ± 1 months old, were maintained on a 14/10 h light/dark (14:10 LD) cycle at 28°C in 3 L tanks of a multitank system (Aquaneering), as per standard practices (Whitmore et al., 2000). They were fed three times per day, at zeitgeber times (ZT) 1, 4, and 9 (ZT0 is lights-on time), with live brine shrimp supplemented with fish pellets (Lansy NRD). All animal procedures were performed in accordance with Institutional Animal Care and Use Committee at Boston University School of Medicine.

##### 5-Bromo-2′-deoxyuridine treatment.

At 2 or 4 h intervals over a 24 h period, individual groups (*n* = 6 per 1 L tank) were treated with S-phase marker 5-bromo-2′-deoxyuridine (BrdU; Sigma-Aldrich), with stock solution administered directly into a 1 L fish tank. The choice of this BrdU dose (6.5 mm) was based on a series of preliminary experiments, using a range of BrdU concentrations in tank water and times of exposure, and compared with an intraperitoneal injection of 10 mm BrdU solution (l μl/100 mg body weight), as per previous studies (Zupanc et al., 2005). Our preliminary data also indicated that a 1-h-long immersion in 6.5 mm BrdU solution was sufficient to reliably label the brain cells in S phase *in vivo*. It was taken into consideration that previous zebrafish studies did not reveal any short-term toxicity of higher BrdU doses used for immersion (Byrd and Brunjes, 2001; Laranjeiro et al., 2013; Makantasi and Dermon, 2014), with this being supported by our survival studies in larval and adult fish (not reported here). We also relied on previous reports that the BrdU method is not sensitive enough to detect DNA repair, suggesting that BrdU-labeled cells represent a population undergoing S phase of CDC (Palmer et al., 2000; Cooper-Kuhn and Kuhn, 2002). This was also supported by the presence of the BrdU+ cells only in the earlier identified neurogenic niches and not other regions of zebrafish brain where DNA repair might occur.

##### Palbociclib (PD 0332991) treatment.

A selective inhibitor of cyclin-dependent kinase (CDK) 4/6, PD 0332991 (Selleckchem.com), was administered directly into the tank water. The dose (2 μm final concentration in tank water) was based on previous reports of dose dependence of the *in vitro* and *in vivo* effects of PD 0332991 suggesting efficacy for concentrations as low as 0.08 μm and an exclusive G_1_ arrest being maintained at concentrations as high as 10 μm (Fry et al., 2004), with cytotoxicity not observed at levels below 5 μm (Barton et al., 2013). Fish were exposed to the drug for 8 h, from ZT22 (Night 1) to ZT6′ (′ is next day), followed by a washout period until sample collection, to cover the entire period of G_1_/S transition in the neurogenic niches studied. To determine whether the drug acutely changes the expression of clock genes or genes involved in cell cycle progression, fish were collected during treatment (ZT3). To document the delayed consequences of PD 0332991 administration on CDC progression, fish were collected on Night 2 at ZT19′ or ZT23′ for the analysis of the expression of CDC regulators. For immunohistochemical analysis of the effects of PD 0332991 on the number of cells undergoing S phase, fish were treated with BrdU for 4 h before sample collection at ZT11′ or ZT15′ (Night 2), i.e., at the time of peak in S-phase pattern in all the neurogenic niches studied (*n* = 5–6 per treatment-by-time group).

##### Evaluation of intrinsic circadian rhythms in constant darkness.

Fish were habituated within experimental groups (*n* = 6 per 3 L tank) for 1 week in 14:10 LD and transferred as a group into nontransparent 1 L tanks at the beginning of the dark phase (ZT14 or beginning of Night 1). Fish were then maintained in constant darkness (DD), for 37–69 h, on a recirculating multitank system. Sample collection for the analysis of changes in mRNA abundance for clock genes and genes involved in cell cycle progression was initiated at ZT3′ on subjective Day 2 (37 h in DD) and proceeded every 4 h for the next 32 h (i.e., until 69 h in DD). The matched control groups remained in LD, and food was provided simultaneously to control and DD-exposed animals.

##### Immunohistochemistry.

Fish were killed with an overdose of MS222 (200 mg/l), and heads were fixed overnight in 4% paraformaldehyde in PBS at 4°C. Brains were then dissected out, cryoprotected in 30% sucrose/PBS, placed in embedding solution (O.C.T.), and stored at −80°C until cut. Coronal 20 μm sections were prepared using a cryostat (Microm HM505E), placed onto Fisherbrand Superfrost Plus slides (Thermo Fisher Scientific), and stored at −80°C until processed. The sections were washed in 0.1 m PBS, and antigen retrieval was performed using both heat, incubated in 50% formamide/50% 2× SSC at 65°C for 2 h, and acid, 2 m HCl at 37°C for 30 min and 0.1 m boric acid, pH 8.5, for 10 min at room temperature. After a PBS wash, the slides were incubated overnight in primary rat anti-BrdU antibody (1:500, Accurate; all antibodies in 0.1 m KPBS plus 0.4% Triton X-100) at 4°C. Then, the slides were incubated for 1.25 h in secondary biotinylated rabbit anti-rat (1:1000, Vector Laboratories). After washing in PBS, chromogenic visualization was accomplished using the Vectastain Standard ABC kit (Vector Laboratories), with NiDAB (Sigma-Aldrich) as a substrate. After rinsing in PBS, sections were dehydrated in an alcohol series, cleared with xylene, and mounted with Permount (Thermo Fisher Scientific). For fluorescence immunohistochemistry, following both antigen retrieval steps, the slides were exposed to primary antibodies for rabbit anti-pH3 (1:1000, Millipore) and rat anti-BrdU (1:1000, Accurate) overnight at 4°C. After washing in PBS, the slides were incubated for 1 h in secondary donkey anti-rabbit Alexa Fluor 555 (1:1000, Invitrogen) and donkey anti-rat Alexa Fluor 488 (1:1000, Invitrogen). The sections were washed in PBS and mounted using Vectashield mounting medium (Vector Laboratories).

##### Microscopy and analysis.

Light microscopy images were taken with a Zeiss Axioskop microscope equipped with a Q Color 5 Olympus camera, 20× objective, and Q Capture Images software. Confocal microscopy was performed with a Zeiss LSM 710 using the Observer Z1 inverted microscope. Using Zen software, images were taken with a 20× objective. To minimize cross talk between channels in multicolored tissue, sequential image acquisition was performed. The absolute number of labeled cells was quantified in the entire dorsal telencephalic, preoptic area, habenular, hypothalamic, and cerebellar neurogenic niches (according to Grandel et al., 2006). To account for interindividual difference in brain size, the data were adjusted for brain volume, which was estimated based on images of individual brain sections (ImageJ) and using the Cavalieri principle (Prakash et al., 1994). Statistical significance for mean values was assessed with an unpaired Student's *t* test, with a significance level of *p* < 0.05.

##### Real-time quantitative RT-PCR.

At each time point, groups of fish were submerged in liquid nitrogen. Brains were dissected out on dry ice and stored at −80°C until analysis. RNA extraction from brain tissue was conducted using QIAzol Lysis Reagent (Qiagen) before RNA isolation using the RNAeasy kit (Qiagen). The quantity and quality of RNA was determined spectrophotometrically at 260/280 nm. The same amount of RNA from each sample was converted into cDNA using the High-Capacity cDNA Archive kit (Applied Biosystems) according to the manufacturer's instruction. Quantitative RT-PCR (qPCR) was performed using a TaqMan Universal PCR Master Mix or SYBR Green PCR Master Mix and ABI Prism 7300 Real Time PCR System (Applied Biosystems). The TaqMan probes and SYBR Green primers were based on the following sequences: cyclin D1 (forward: 5′-GTC GCG ACG TGG ATG C-3′, reverse: 5′-CCA GGT AGT TCA TAG CCA AAG GAA A-3′, {TCG AGG TCT GTG AAG AGC AG}); cyclin E (forward: 5′-AGC GCT TTA GAT TCA AGA ACC TCT T-3′, reverse: 5′-GCT CCT TGT TCA GCA TCT TTA TCC A-3′, {CCA GAC CCC TGT TTA GC}); cyclin A2 (forward: 5′-TGG AGA ACA ACC AGA GGA GAC A-3′, reverse: 5′-GCA TTT TCT TCA GGT TTA CAC GCA AT-3′, {CCA GAC CCC TGT TTA GC}); cyclin B2 (forward: 5′-GCG AAC TGT CTA ATC TTT CCC ACA A-3′, reverse: 5′-CGG CCA GTG GGT TTT ACA C-3′, {CAG TTC AGA CAA AGA AGG TT}); *Bmal1* (forward: 5′-CAG AGC TTC GCC ACA AAC C-3′, reverse: 5′-CTG TGA TCA ATG CAT GTC CTT TCA-3′, {CTCGATGTGAGGATCTG}); *Clock1* (forward: 5′-CAT CCT ACA GAA GAG CAT CGA CTT-3′, reverse: 5′-GAT TTC ACT CGA CTC CGA CTG T-3′, {AAG CAC AAA GAA ATT G}); *Per1* (forward: 5′ATT CCG CCT AAC CCC GTA TGT GAC C-3′, reverse: 5′GTG TGC CGC GTA GTG AAA ATC CTC TTG T-3′); *p20* (forward: 5′-GGT CCG TGT GGA CTT GAT TT-3′, reverse: 5′-CCT CTT CAA CAG CCC ATG AT-3′); *Wee1* (forward: 5′-CAT CGC CAC GGA AAG TC-3′, reverse: 5′-TGG GGG GTA TCA AAA AGA C-3′); *p21* (forward: 5′-CCG CAT GAA GTG GAG AAA AC-3′, reverse: 5′-ACG CTT CTT GGC TTG GTA GA-3′). Gene expression was normalized for two housekeeping genes: β-actin and *EF1a* expression level for each individual fish sample: β-actin (forward: 5′-GCT GTT TTC CCC TCC ATT GTT G-3′, reverse: 5′-TTT CTG TCC CAT GCC AAC CA-3′; {CCC AGA CAT CAG GGA GTG}); *EF1a* (forward: 5′-GCA CGG TGA CAA CAT GCT-3′, reverse: 5′-TCC TTG CGC TCA ATC TTC CAT-3′; {ACC AGC CCA TGT TTG AG}). Relative mRNA expression level was calculated using the standard comparative [Delta]-Ct method.

##### Statistical modeling of circadian kinetics of S-phase entry and S length.

For a given neurogenic niche, the distribution of phase angles of S-phase initiation within a given day *G*(*t*) was modeled as a Gaussian function with a mean μ, dispersion σ, multiplied by a rate scaling constant *r*. The length of the S phase was modeled as a constant value of ε. The resulting number of BrdU-labeled cells *n*(*t*_0_), measured at a time *t*_0_, was then modeled as a sum of all cells that were within the S phase during BrdU labeling. To account for the circadian periodicity of the signal, the contributions of cells originating from different days (labeled by index *k*) are summed up as follows:

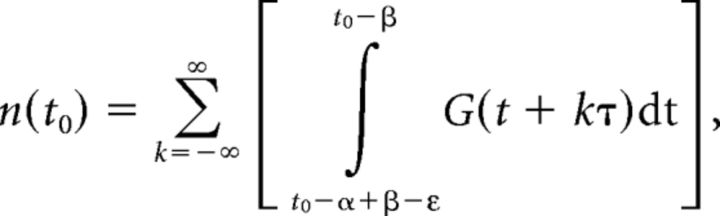
 where α is the length of the BrdU labeling (4 h), β is the minimal amount of time a replicating cell needs to overlap with the BrdU pulse to be reliably labeled (1 h), and τ is the length of the circadian cycle (24 h). Given the numbers of the BrdU-labeled cells observed at different time points, the posterior distributions of the remaining parameters (μ, σ, r, ε) were estimated following a Bayesian approach, using No-U-Turn sampler (Hoffman and Gelman, 2011), as implemented by the RStan package. Prior distribution for μ was taken to be *N*(*12 h, 24 h*). σ Prior was taken to be a Gamma distribution with shape of 2.5 and a rate of 0.5. Prior for *r* was taken to be gamma distribution with shape of 3 and rate of 3/*p*, where *p* is the maximum observed number of BrdU-labeled cells. The prior distribution for ε was taken to be a broad Beta distribution with parameters 1.5 and 1.5 rescaled to the 24 h time period. For computational efficiency, only three adjacent days were taken into account (i.e., only values of *k* equal to −1, 0, and 1 were considered), as contribution of more distant days was found to be negligible. To estimate joint posterior distributions, a total of 5000 sampling iterations were performed.

## Results

### Daytime S-phase progression is common to all neurogenic niches, with niche-specific distribution of the phase angle of S initiation and S length

To characterize circadian variation in the number of cells undergoing S phase in adult brain under entrained light–dark conditions, groups of zebrafish were exposed to BrdU for 4 h, at multiple time points throughout the 24 h cycle. Further evaluation of the number of BrdU-positive cells was conducted in five neurogenic niches, including the dorsal telencephalon (DT), parvocellular preoptic area (PPa), habenula (Hb), hypothalamus (Hyp), and cerebellum (Cer). Of 16 neurogenic niches documented in adult zebrafish and distributed along the whole rostrocaudal brain axis, we chose these five based on previous studies demonstrating either their high level of homology to mammalian structures (Cer, Hb, Hyp, PPa) or homology to specific areas of adult neurogenesis in mammals (e.g., DT being homologous to hippocampus; Zupanc et al., 2005; Kaslin et al., 2009; Cheng et al., 2014). Moreover, Cer and DT represent some of the most active proliferating zones in adult zebrafish brain, with different stem cell types present (Kaslin et al., 2013; Than-Trong and Bally-Cuif, 2015).

**Figure 1.**
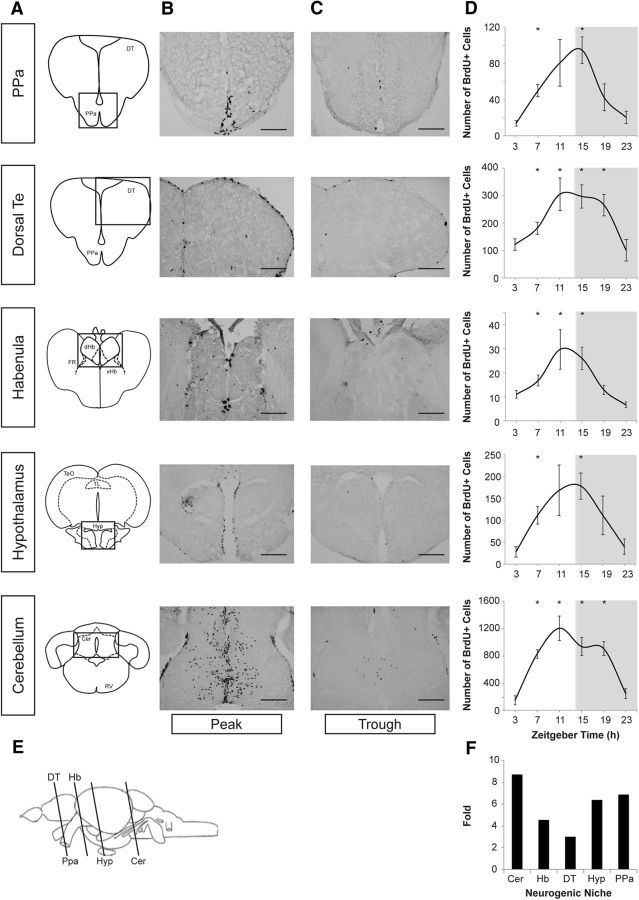
Daily pattern of S phase of the cell division cycle in five neurogenic niches of adult zebrafish brain. ***A***, Schematic of five neurogenic niches of adult zebrafish brain, with boxes identifying the areas presented in ***B*** and ***C***. ***B***, ***C***, Representative images of daily variation in the number of BrdU+ cells in each niche, at daily trough (***B***) and peak (***C***), as presented in ***D***. Scale bars, 50 μm. ***D***, Number of BrdU+ cells undergoing S phase within each neurogenic niche, documented at 4 h intervals over a 24 h period. *n* = 4–6 fish per time point; mean ± SEM; *p < 0.05 relative to the trough in BrdU+ cells for a specific niche. The gray background indicates night, 14:10 LD cycle. Samples were collected at specified ZT, after a 4 h *in vivo* exposure to BrdU. ZT0 is lights-on time. ***E***, Schematic of zebrafish brain identifying coronal planes at which the brain was cut to evaluate BrdU+ cells presented in ***B–D***. ***F***, Range of circadian oscillation, the ratio of the number of BrdU-labeled cells at peak and trough in each niche (folds).

All five niches revealed daily variation in the number of cells undergoing DNA replication (Fig. 1). The number of BrdU-labeled cells, total and at peak, was highest in Cer (1183 ± 178.7, peak), with minimal numbers observed in Hb (30 ± 8.3, peak), reflecting the difference in the overall size and proliferative capacity of these niches (Fig. 1*B–D*). Common to all niches was a robust increase in the number of S-phase cells by midday (ZT7), with a peak in the evening and early-night hours (ZT11–ZT19), and a trough at the end of the night (ZT23; Fig. 1*A–D*). Additional experiments with a more frequent sampling rate over shorter intervals demonstrated a similar number of BrdU-positive cells at trough, between ZT23 and ZT1 (data not shown). Such nonzero values at the troughs indicate that some fraction of dividing cells might be uncoupled from the circadian cycle. However, the magnitude of circadian oscillation significantly exceeded this background level in all examined structures (Fig. 1*F*).

**Figure 2.**
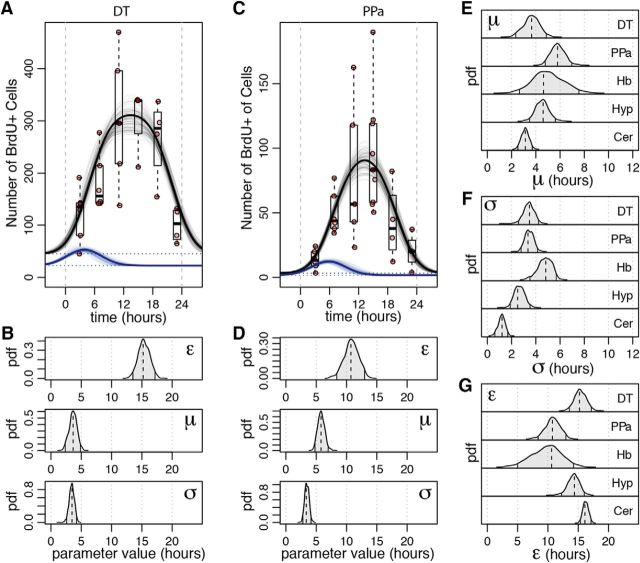
Statistical modeling of circadian kinetics of S-phase progression in diverse neurogenic niches of adult zebrafish brain. ***A***, Boxplots show the number of BrdU+ cells in DT niche, observed at different time points throughout the day. The values for each individual fish are shown as red dots. The black curves show the overall fit of the statistical model (see Materials and Methods) for the observed number of BrdU+ cells. The rate of S-phase entry predicted by the model is shown by blue curves. For both curves, the line shows the maximum likelihood model predictions, and thin lines around it show predictions sampled from the posterior ensemble of the likely model parameter estimates. ***B***, Marginal posterior distributions of the individual model parameters for the DT niche, including mean (μ) and dispersion (σ) of the S-phase entry rate function *G*(*t*) and the length of the S-phase (ε). The shaded region under the curve marks the 95% credible interval. ***C***, ***D***, Fit illustration and posterior distributions for the PPa niche. ***E–G***, Marginal posterior distributions of the μ, σ, and ε parameters for all of the examined neurogenic niches.

To quantify the circadian kinetics of CDC, we constructed a statistical model. From the observed BrdU patterns within each neurogenic niche studied, we modeled the distribution of phase angles of S-phase initiation within a given day as a Gaussian function *G*(*t*) with a mean μ, dispersion σ, and the length of the S phase as a constant ε (see Materials and Methods). Following the Bayesian approach, we evaluated the posterior distributions of these parameters based on the likelihood of attaining the observed cumulative counts of BrdU-labeled cells at different time points throughout the cycle (Fig. 2*A–D*). We found that the mean phase angle of entry into S phase (μ, measured relative to the onset of environmental illumination ZT0) occurs during the first quarter of the day but varies between different niches (Fig. 2*E*), with PPa showing the latest onset (maximum likelihood estimate, 5.8 h) and Cer the earliest (3.1 h). Similarly, neurogenic niches showed significant variation in the degree of synchrony of the S-phase entry by individual cells, as captured by the phase angle dispersion σ (Fig. 2*F*), with Hb showing significantly higher σ (lower synchrony) than all other niches (*p* = 0.035). Finally, the average length of the S phase (ε) also showed notable variation between the niches (Fig. 2*F*), with ε predicted for DT (15.2 h) and Cer (16.1 h) being significantly longer than that for PPa (10.7 h) or Hb (10.6 h; *p* = 0.011).

These results indicate that although circadian dependency of cell proliferation rates is pronounced across all of the examined niches, the prevalent timing of S-phase initiation, the degree of synchronization between the cells, and the length of the S phase itself varies significantly between the neurogenic niches.

### Nighttime progression of cells through G_2_/M phase in adult neurogenic niches

**Figure 3.**
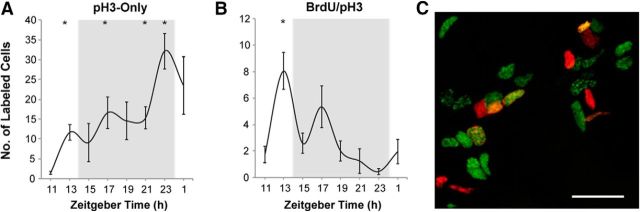
Nighttime transition of dividing cells through G_2_/M phases of the cell cycle in the cerebellum of adult zebrafish. ***A–C***, Number of cells expressing pH3, a late G_2_ and early M phase marker, and BrdU, a marker for S phase. ***A***, Cells positive for pH3-only increase throughout the night, ***B***, Cells with colocalized BrdU/pH3 label decline throughout the night. *n* = 3–6 fish per time point; mean ± SEM; *p < 0.05 relative to the trough in labeled cells (pH3-only, ZT11; BrdU/pH3, ZT23). The gray background indicates night, 14:10 LD cycle. Fish were collected at specified ZT, after a 2 h *in vivo* exposure to BrdU. ZT14 is lights-off time. ***C***, Representative confocal image: cells labeled for pH3-only (red), BrdU-only (green), and BrdU/pH3 merge (yellow). Scale bar, 20 μm.

We used the phosphorylation of histone H3 (pH3) at serine 10, a relatively short-lived marker present specifically in late G_2_ and early M phases (Hendzel et al., 1997), to determine the daily pattern of G_2_/M in individual neurogenic niches. Daily variation in pH3-positive cells was first evaluated at 4–6 h intervals, suggesting predominantly nighttime expression (data not shown). This was further confirmed using higher temporal resolution, with pH3 being documented at 2 h intervals (Fig. 3*A*,*B*). The increase in pH3 labeling was observed throughout the night, with a peak at ZT23. This was consistent with the trough in BrdU-labeled cells at that time (Fig. 1*D*), confirming that by the end of the night, the majority of dividing cells completed the S/G_2_ transition, proceeding to mitosis.

Although all the fish were exposed to BrdU for 2 h before sample collection, in some cells only pH3 could be documented, whereas others were colabeled for pH3 and BrdU (Fig. 3*A*,*B*). For cells to express only pH3, they had to exit S phase at least 2 h before documented expression of the G_2_/M marker (Fig. 3*A*). In contrast, those colabeled for BrdU and pH3 had to accomplish both S and at least part of G_2_ within the 2 h interval of BrdU exposure, indicating that the length of their G_2_ until the time of pH3 expression was substantially shorter than 2 h (Fig. 3*B*). The number of such double-labeled cells declined overnight. Together, this suggested a potential difference in G_2_ length in cells within the same neurogenic niche and an increase in G_2_ length overnight.

### Entrained and intrinsic rhythms of mRNA expression for cell cycle regulators and core clock genes in adult zebrafish brain

**Figure 4.**
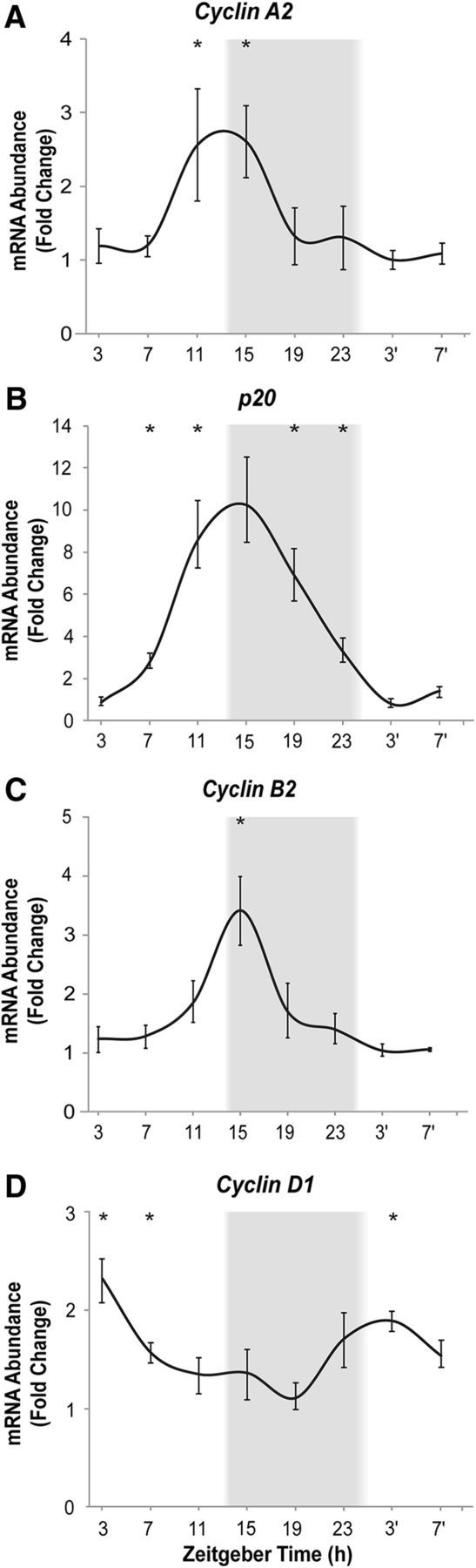
Daily cycle of mRNA expression for cell cycle regulators in adult zebrafish brain. Shown is transcript expression for cyclin A2 (***A***), *p20* (***B***), cyclin B2 (***C***), and cyclin D1 (***D***). *p < 0.05 relative to the time of trough for mRNA abundance for each gene (ZT3′ for cyclin A2, p20, cyclin B2, and ZT19 for cyclin D1). *n* = 5–6 fish per time point; mean ± SEM. The gray background indicates night, 14:10 LD cycle. ZT of sample collection, ZT0 is lights-on time.

**Figure 5.**
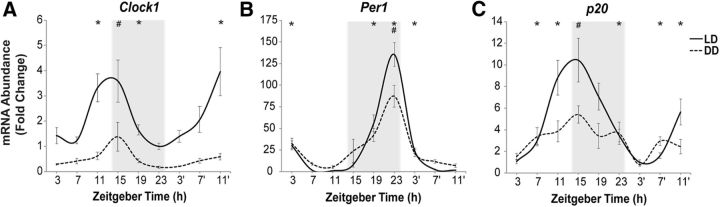
Intrinsic and entrained circadian variation in mRNA abundance for p20 and core clock genes. Shown are circadian patterns of mRNA abundance for *Clock1* (***A***), *Per1* (***B***), and *p20* (***C***) under LD (solid line) and DD (dashed line) conditions. *n* = 5–6 fish per time point; mean ± SEM. The gray background indicates night, 14:10 LD cycle. ZT of sample collection, ZT0 is lights-on time. Intrinsic circadian rhythm in DD, *p < 0.05, relative to the time of trough for mRNA abundance for each gene in DD (ZT23 for Clock1, ZT11 for Per1, ZT3′ for p20). Reduction in mRNA abundance at peak, DD versus LD, ^#^*p* < 0.05 (ZT15 for Clock1 and p20, ZT23 for Per1).

To further explore the timing of the cell cycle phase transitions in adult brain and their correlation with the core circadian clock machinery, the mRNA expression patterns for critical cell cycle and circadian clock regulators were assessed using qPCR (Fig. 4). We analyzed expression of cyclins involved in G_1_/S transition (D1 and E), S progression (A2), and G_2_/M (B2; for review, see Stacey, 2003). We also documented expression patterns for cyclin-dependent kinase inhibitor p20 (CDK-p20) that limits transition between the G_1_ and S phases (Laranjeiro et al., 2013). Two genes representing the positive and negative parts of the feedback loop of the clock, respectively, were *Clock1* and *Per1* (Fig. 5).

The expression of cyclin A2 was increased throughout late day and into the night hours (Fig. 4*A*). The expression of *p20* peaked at approximately the same time, with acrophase around ZT11–ZT19 (Fig. 4*B*), preceding robust decline in the number of S-phase cells (Fig. 1). The expression of cyclin B2 also peaked at day–night transition (Fig. 4*C*). Thus, late-day and early-night upregulation of mRNA abundance involved genes critical for the middle and end of the cell cycle, i.e., S-phase progression and G_2_/M. In contrast, the expression of cyclins involved in the early phases of the cell cycle occurred in the morning. The expression of cyclin D1, which controls progression through late G_1_ phase, was increased by the end of night and peaked in the early-morning hours, at ZT3 (Fig. 4*D*). The expression of cyclin E1, involved in G_1_/S checkpoint transition (Ohtsubo et al., 1995), typically surged close to the cyclin D peak time, but its pattern was highly inconsistent between independent experiments, in contrast to other cyclins (data not shown). We assessed two other cell cycle regulators in the zebrafish brain, *Wee1* and *p21*, known to be under clock control in other tissues (Matsuo et al., 2003; Grechez-Cassiau et al., 2008; Laranjeiro et al., 2013), but those displayed very low mRNA abundance in zebrafish brain, with no indication of daily variation present (data not shown). Similar to what we have reported previously (Zhdanova et al., 2008), the *Clock1* transcript peaked at the end of the day (ZT11–ZT15), reaching trough by the end of the night at ZT23 (Fig. 5*A*), whereas *Per1* mRNA expression in zebrafish brain reached its peak late at night at ZT23 (Fig. 5*B*).

To determine whether the observed daily rhythms of expression for the principal cell cycle regulators reflect intrinsic circadian oscillations, animals were studied in DD. The mean circadian variation for the core clock genes, while studied in LD, was 135-fold for *Per1* and 3.6-fold for *Clock1*. After 48 h in DD, the acrophase for the clock genes remained similar to that in LD, whereas the magnitude of variation for *Clock1* and *Per1* was significantly reduced (Fig. 5*A*,*B*). This was anticipated based on previous reports in diverse species of entrained rhythms of activity or gene expression having significantly higher amplitude when compared with the intrinsic rhythms (Tamai et al., 2005; Huang et al., 2011; Hughes and Piggins, 2014; Ramkisoensing and Meijer, 2015). Moreover, a desynchronization between the fish with different intrinsic circadian periods can be expected in the absence of entrainment to light, further reducing mean group amplitude. No significant circadian variation in cyclin patterns could be documented in DD (data not shown), potentially because of their low baseline circadian variation in LD (twofold to threefold; Fig. 4), comparable with the degree of reduction in clock gene expression between entrained and constant conditions. In contrast, a higher magnitude of daily variation in *p20* transcript expression with a peak of 10.4 ± 2.0-fold, though also substantially decreased, retained significant intrinsic circadian variation in DD (Fig. 5*C*). Together, this suggested presence of intrinsic CDC rhythm, with entrainment to light enhancing circadian oscillations in adult neurogenesis.

### The G_1_/S transition in the morning hours enables progression of the cell cycle over the following 24 h period

Based on a combination of the observed daily expression patterns for cyclins and *p20*, daytime increase in S-phase progression (as per BrdU), and late G_2_/M phase peaking by the end of the night (as per pH3), we hypothesized that upregulation of cyclin D expression throughout the morning reflects the timing of the G_1_/S transition for the cells undergoing division over the next 24 h period. We thus predicted that interfering with cyclin D effects at the end of Night 1 and in the morning hours would reduce BrdU labeling throughout the day and expression of cyclins A2 and B2 on Night 2, reflecting a diminished population of cells progressing through S and G_2_/M earlier in the day.

**Figure 6.**
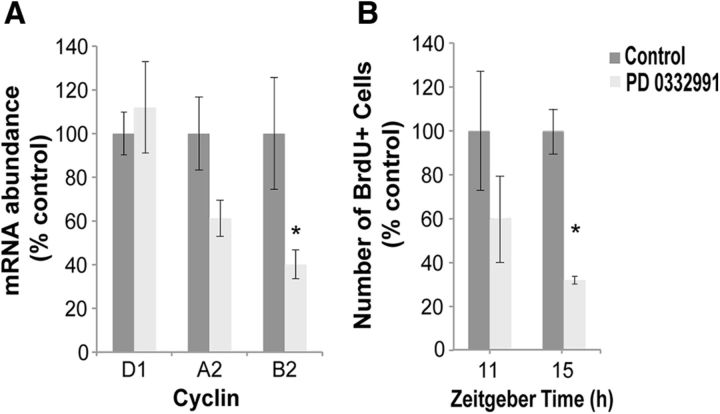
Interference with cyclin D function on Night 1 inhibits S- and G_2_/M-phase progression over the following day and Night 2. ***A***, Administration of PD 0332991, a CDK4/6 inhibitor, at ZT22 of Night 1 leads to a reduction in the expression of cyclins A2 and B2 on Night 2 (ZT19′ and ZT23′, combined). ***B***, Reduction in the number of BrdU+ cells during the day (ZT11′) and Night 2 (ZT15′), after a 4 h exposure to BrdU (immersion). *n* = 5–6 fish per time point; mean ± SEM; *p < 0.05 relative to control. ZT0 is lights-on time.

To test this hypothesis, we used PD 0332991, a highly selective inhibitor of the cyclin D kinases CDK4 and CDK6, which blocks retinoblastoma protein phosphorylation, inducing G_1_ arrest independent of the presence of functional D1 cyclins in the cell (Fry et al., 2004). The inhibitor was administered starting the end of Night 1 (ZT22), when mRNA expression for cyclin D1 surges. Fish were then continuously exposed to the drug until ZT6′, to cover the period of increased D1 transcript expression and rapid increase in the number of BrdU-positive cells, requiring cyclin-activated D1 kinases to move cells toward the G_1_/S checkpoint. Such alteration in the cyclin D1-regulated pathway did not interfere with the expression levels for the cyclins on Night 1 or early morning, suggesting lack of acute effect on cyclin production (data not shown). Importantly and consistent with our hypothesis, the treatment led to the reduced expression of cyclins A2 and B2 on Night 2, with expected lack of changes in cyclin D1 expression (Fig. 6*A*). This was also supported by significantly reduced BrdU labeling on Night 2 (Fig. 6*B*). Together, this indicated that G_1_ arrest on Night 1 and in the morning alters transition of cells through S phase later that day and their progression toward mitosis on Night 2, thus supporting our hypothesis.

## Discussion

**Figure 7.**
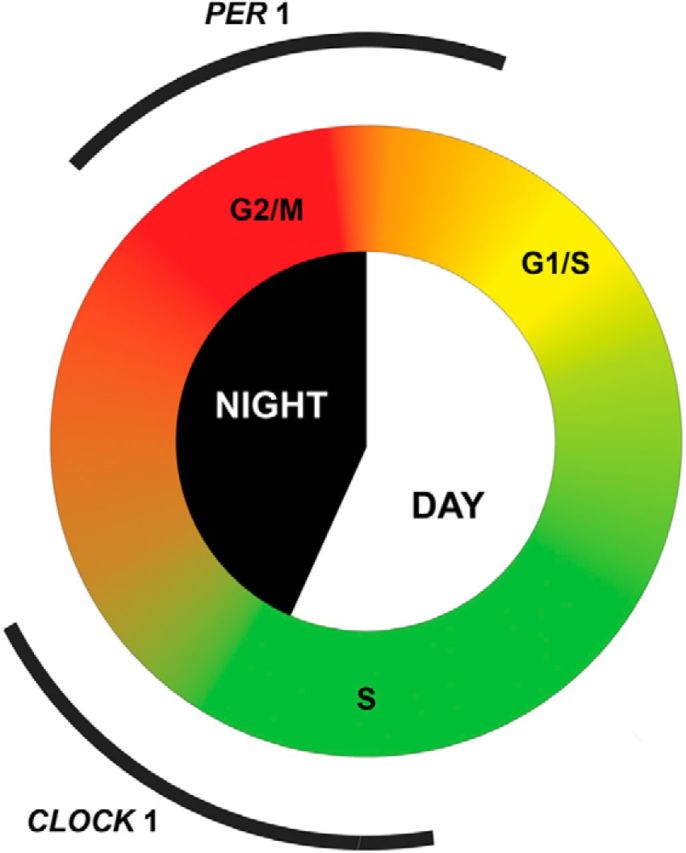
Schematic of the typical cell cycle progression in adult brain of a diurnal vertebrate, zebrafish, maintained under regular light–dark conditions. At early-morning hours of Night 1, upregulation of cyclin D1 expression initiates gradual G_1_/S transition in the population of cycling neuronal stem and progenitor cells. Throughout the day, S-phase initiation and DNA replication continues, with a gradual surge in cyclin A2 expression and the number of S-phase cells peaking late in the day. The surge in *p20* expression is initiated by the end of the day, limiting further S-phase initiation. These CDC events are coupled to the activation of the positive feedback loop of the circadian clock, as per Clock1 transcript levels. Cyclin B2 expression is upregulated thereafter, promoting late G_2_ and M phases of the cell cycle throughout Night 2, with the cell cycle culminating by the end of the night. The end of the cycle and the preparation for the next G_1_/S transition is coupled to the initiation of the negative feedback loop mechanisms of the clock, coinciding with upregulation of Per1 transcription at the end of the night.

This study establishes that, in diverse adult neurogenic niches of a diurnal vertebrate, cell cycle progression displays robust entrained circadian rhythm. Starting in the morning, cells gradually transit from G_1_ to S phase of the cell cycle. This process spreads over several hours, with a peak number of cells undergoing S phase observed close to the time of light–dark transition. Thereafter, new cells are prevented from entering S phase, at least in part attributable to activation of the cyclin-dependent kinase inhibitor p20 around that time. This is followed by cells gradually exiting S and transiting into G_2_/M phase throughout the night, with peak in G_2_/M observed by the end of the night (Fig. 7).

Under stable environmental conditions in the presence of an environmental light–dark cycle, CDC is coupled to the circadian clock rhythm in zebrafish brain (Fig. 7). The peak in S phase corresponds to the activation of the positive arm of the circadian loop, as per expression pattern for *Clock1* mRNA. The CDC is completed at the time of upregulation of *Per1* mRNA expression, toward late-night and early-morning hours. Preservation of circadian variation in transcript levels for a prominent CDC regulator, *p20 (*Fig. 5*C*), in the absence of entraining environmental photoperiod also suggests that the circadian pattern of CDC in zebrafish brain can be intrinsic in nature.

Several previous reports implied that phase relationship between the clock and CDC in diverse tissues can be primarily conserved between species and preserved *in vitro*. In cultured human fibroblasts, despite a much shorter than 24 h period, the peak of expression for Rev-erb transcript that, similar to *Per1*, reflects a negative feedback loop of the clock is coupled to the G_1_/S transition (Feillet et al., 2015). Also consistent with our results, the *p20* transcript levels peak at day–night transition in zebrafish cell lines, embryos, and adult brain tissue (Laranjeiro et al., 2013). Interestingly, in night-active mice, cell proliferation in the subgranular zone (SGZ) of adult hippocampus is also increased at night (Bouchard-Cannon et al., 2013). However, it shows substantial delay in the time of initiation, relative to the typical timing of S-phase entry we find in day-active zebrafish, and higher background levels. Whereas our data demonstrate a consistent pattern of circadian variation in CDC progression across all examined zebrafish neurogenic niches, there is lack of such consistency in mice. The surge in the number of M-phase cells in SGZ at nighttime is in contrast with lack of such circadian variation in the second neurogenic niche in mice, the subventricular zone of the forebrain, and no significant variation in S phase observed in either region in these animals (Tamai et al., 2008). Further in-depth investigation into the differences in circadian regulation of neurogenesis between diurnal and nocturnal species could be of principal importance for translational research, in view of the inverse relationship between the central clock and downstream clock-controlled processes in these animals.

We conducted comparison between CDC patterns documented in individual neurogenic niches of zebrafish brain. Since BrdU labeling provides cumulative tallies of the number of cells undergoing divisions, we relied on statistical modeling to estimate the rate at which the cells in different neurogenic niches enter the S phase throughout the 24 h period. The results confirm that circadian dependency of cell proliferation rates is pronounced across all of the examined niches. They also reveal significant differences between the neurogenic niches, with respect to the magnitude of circadian oscillation, prevalent timing of S-phase initiation (phase angle), the degree of synchronization between the cells, and the extent of the S-phase length. For example, the cerebellar niche (Cer), the largest in zebrafish brain, has a high magnitude of circadian oscillation, reflecting relatively low numbers of S-phase cells at day–night transition and thus fewer cells that are not following the prevalent temporal pattern of cell cycle progression. Cer is also the niche with the most advanced phase angle of S entry, corresponding to a more synchronized G_1_/S-phase transition among dividing cells. In contrast, another relatively large neurogenic niche located in DT has a low magnitude of daily oscillation, with a high number of cells not following the typical circadian pattern of CDC. The DT also has late S-phase onset and low synchronization between cells. This is despite both Cer and DT niches having similar mean S-phase length, substantially longer than in other niches studied.

These results raise intriguing questions of whether interniche differences in cell cycle progression might reflect a different strength of central circadian regulation in these brain regions. Presence of local niche-specific factors, related to diverse physiological functions and neuronal connections of these brain regions, might also synchronize cells in their transition through CDC in a niche-dependent way. Similarly, the difference in the mean length of S phase suggested by our model might reflect homogenous populations of niche-specific cells or a different proportion of cells with longer and shorter S-phase length in each niche, as shown in some previous studies (Brandt et al., 2012; Feillet et al., 2015). Moreover, our data on the presence or absence of coexpression of S- and G_2_/M-phase markers (BrdU and pH3) over consecutive 2 h intervals also suggest that the length of G_2_ can vary between cells, increasing overnight.

It remains to be seen which cell qualities could reflect different CDC kinetics. For example, the timing at which dividing cells transit G_1_/S and the length of their S or G_2_ phases might differ between actively self-renewing multipotent neural stem cells (NSCs) and amplifying neural progenitor cells (NPCs) with a limited number of divisions (Grandel et al., 2006). The known NSC heterogeneity and plasticity (Kaslin et al., 2013; Than-Trong and Bally-Cuif, 2015; Barbosa and Ninkovic, 2016) might also contribute to the variation in circadian patterns of CDC progression. It will be important to determine whether adhering to a well-synchronized circadian pattern of cell division might help in maintaining maintain a healthy pool of specific NSCs with broad potential or NPCs with restricted potential within adult neurogenic niches.

The timing of G_1_/S transition, the degree of synchrony between the cells of the same niche in entering S, or the length of S and G_2_/M phases might also correlate with the survival, migration, or neuronal fate of new cells in adult brain. This first study on the circadian variation of CDC in neurogenic niches of adult zebrafish brain did not aim at addressing these issues. However, additional studies, requiring extensive pulse-chase investigation initiated at different times of day, with cells being followed by several neuronal and glial markers over prolonged periods of time, could establish the extent to which the timing of cell progression through S or M phases might affect cell fate. Previously, it was reported that the vast majority of dividing cells in adult zebrafish brain give rise to neurons, with only a minority of cells differentiating into glia (Grandel et al., 2006; Kaslin et al., 2009, 2013). This and only a small number of cells replicating outside the typical circadian cycle, as per the background S-phase cells we observed late at night and early in the morning, suggest that the circadian pattern of CDC in neurogenic niches reported here is likely to reflect circadian regulation of adult neurogenesis in zebrafish brain.

To summarize, the robust circadian variation in the transition of cells through CDC within adult neurogenic niches demonstrated here using a diurnal vertebrate model suggests that a similar phenomenon might be present in the brain of day-active humans. Understanding the circadian phase of entrainment of CDC phases to environmental and endogenous factors, or time-dependent response of adult neurogenesis to pharmacological interventions, could be of major clinical significance. The timing of such interventions, aimed at either enhancing neurogenesis in brain trauma or neurodegenerative disorder patients or preserving normal neurogenesis during cancer cytostatic therapies, could be critical. Considering the advantages of the model, the search for local niche-specific factors that can modulate daily CDC progression in zebrafish brain may lead to novel prophylactic and treatment strategies.
